# Assessing high-risk sexual practices associated with human immunodeficiency virus infection among young female sex workers in Lubumbashi, Democratic Republic of the Congo: a cross-sectional study

**DOI:** 10.1186/s12981-024-00602-x

**Published:** 2024-03-19

**Authors:** Olivier Mukuku, Yannick Nkiambi Kiakuvue, Georges Yumba Numbi, Bienvenu Mukuku Ruhindiza, Christian Kakisingi, Claude Mulumba Mwamba, Joe Kabongo Katabwa

**Affiliations:** 1 Institut Supérieur des Techniques Médicales de Lubumbashi, Lubumbashi, Democratic Republic of the Congo; 2 Faculty of Medicine, University of Lubumbashi, Lubumbashi, Democratic Republic of the Congo; 3 Faculty of Medicine, University of Ngozi, Ngozi, Burundi

**Keywords:** HIV, Young female sex worker, Prevalence, Risky sexual practices, Lubumbashi

## Abstract

**Introduction:**

Young female sex workers (YFSWs) face a higher risk of HIV infection compared to older workers, but there is a lack of comprehensive data on their sexual practices and HIV infection risks, which may present unique challenges and vulnerabilities. The study aimed to identify high-risk sexual practices associated with HIV infection among YFSWs in Lubumbashi.

**Methods:**

We conducted an analytical cross-sectional study and used a comprehensive sample of all YFSWs who presented to the HIV/Sexually Transmitted Infections Screening and Treatment Center in Lubumbashi between April 2016 and December 2017. We collected data on socio-demographic characteristics and behavioral risk factors of female sex workers were collected using a structured questionnaire. Using STATA version 16, multivariate logistic regression was fitted and the results were presented as adjusted odds ratios (aORs) with their 95% confidence intervals (95% CIs).

**Results:**

A total of 572 YFSWs were included in the study, 19 of whom were HIV-positive (3.3%; 95% CI: 2.1–5.1%). Participants who were forced to have sex (aOR = 12.2; 95% CI: 3.2–46.4; *p* < 0.0001), those who did not use condoms systematically (aOR = 4.1; 95% CI: 1.3–13.0; *p* = 0.018), and those who had anal sex (aOR = 23.8; 95% CI: 6.9–82.4; *p* < 0.0001) were more likely to be HIV-positive.

**Conclusion:**

The study reveals a concerning trend of higher hospital HIV prevalence among YFSWs compared to the general Congolese population. It also highlights a significant link between high-risk sexual practices and HIV infection, highlighting the need for urgent interventions.

**Table:** 

Contributions to the Literature
• Young female sex workers (YFSWs) in Lubumbashi face a higher risk of HIV infection compared to the general population.
• This study identifies key factors contributing to their vulnerability, including forced sex, non-condom use, and engaging in anal sex.
• This research contributes vital insights for public health strategies, advocating for tailored initiatives to address the specific challenges faced by YFSWs in preventing HIV transmission.
• By shedding light on the nexus between risky sexual practices and HIV prevalence, this study underscores the importance of proactive measures to protect the health and well-being of YFSWs in the fight against HIV/AIDS.

## Introduction

In sub-Saharan Africa, adolescent girls and young women (AGYW) aged between 15 and 24 are at a higher risk of being exposed to human immunodeficiency virus (HIV) infection than older women and their male peers. This is particularly true for those who sell sex, who are highly vulnerable to HIV transmission [1–3]. Sex work’s precarious conditions expose vulnerable populations, particularly young female sex workers (YFSWs), to increased HIV risk, a major public health concern, particularly among AGYW in diverse socio-cultural settings, highlighting the need for improved health care [4]. Economic pressures, customer retention problems, and forced sex are just a few of the factors that YFSWs must navigate, which make engaging in risky sexual practices more likely [5]. Studies have shown that compared to older FSWs, YFSWs negotiate condom use less, are more likely to have sex with older partners, and are less likely to benefit from HIV prevention and other health services due to their fear of stigmatization and discrimination by medical staff [6–9]. Sarkar et al. [10] found that HIV infection among YFSWs was 2.4 times higher than in older FSWs, as YFSWs’ professional immaturity may lead to more unprotected sex [10]. A Chinese study by Su et al. [11] confirmed that AGYW new to sex work are more likely to be victims of physical and sexual abuse, which can double or quadruple HIV prevalences. The stigma surrounding AIDS and sexuality as well as ignorance of the use of HIV post-exposure prophylaxis (PEP) after forced sex add a layer of complexity to understanding the risk factors involved in HIV transmission [12].

Despite the well-known risks associated with YFSWs, it is important to understand the high-risk sexual behaviours associated with HIV transmission to develop effective interventions and policies aimed at reducing the spread of HIV and improving the health and well-being of FSWs.

The HIV and AIDS epidemic in the DRC is considered a mixed health public problem due to its varying impact on the different provinces. According to the Democratic Republic of the Congo (DRC)’s HIV and AIDS programme’s 2021 estimate, 0.7% of adults aged 15–49 is HIV-positive [13]. HIV prevalence rates vary considerably from province to province, with Maniema showing the highest rates (4%); Haut-Katanga province recorded 1.5% [14]. FSWs consistently have a higher HIV prevalence compared to the general population, with 8.2% of them being HIV-positive in a study conducted in Lubumbashi [15]. If not properly managed, the HIV prevalence among FSWs could increase among the general population due to interactions with FSWs. It is important to note that although previous research has explored the sexual practices and behaviors of various populations, including sex workers, much of this work has focused on adult FSWs [15–17]. There is a lack of comprehensive data on the sexual practices and HIV infection risks specifically among YFSWs, who may face distinct challenges and vulnerabilities compared to older sex workers. There is limited research that examines the sexual behaviors of YFSWs in Lubumbashi. Therefore, it is imperative to conduct this study, which aimed to identify high-risk sexual practices associated with HIV infection among YFSWs in the bustling city of Lubumbashi in southeastern DRC. This research seeks to understand the HIV/AIDS situation in a vulnerable population, YFSWs, by examining the link between risky sexual practices and HIV prevalence. It provides insights for public health strategies to address challenges, prevent HIV transmission, and improve sexual health and well-being among YFSWs.

## Materials and methods

### Study setting

**Fig. 1:**
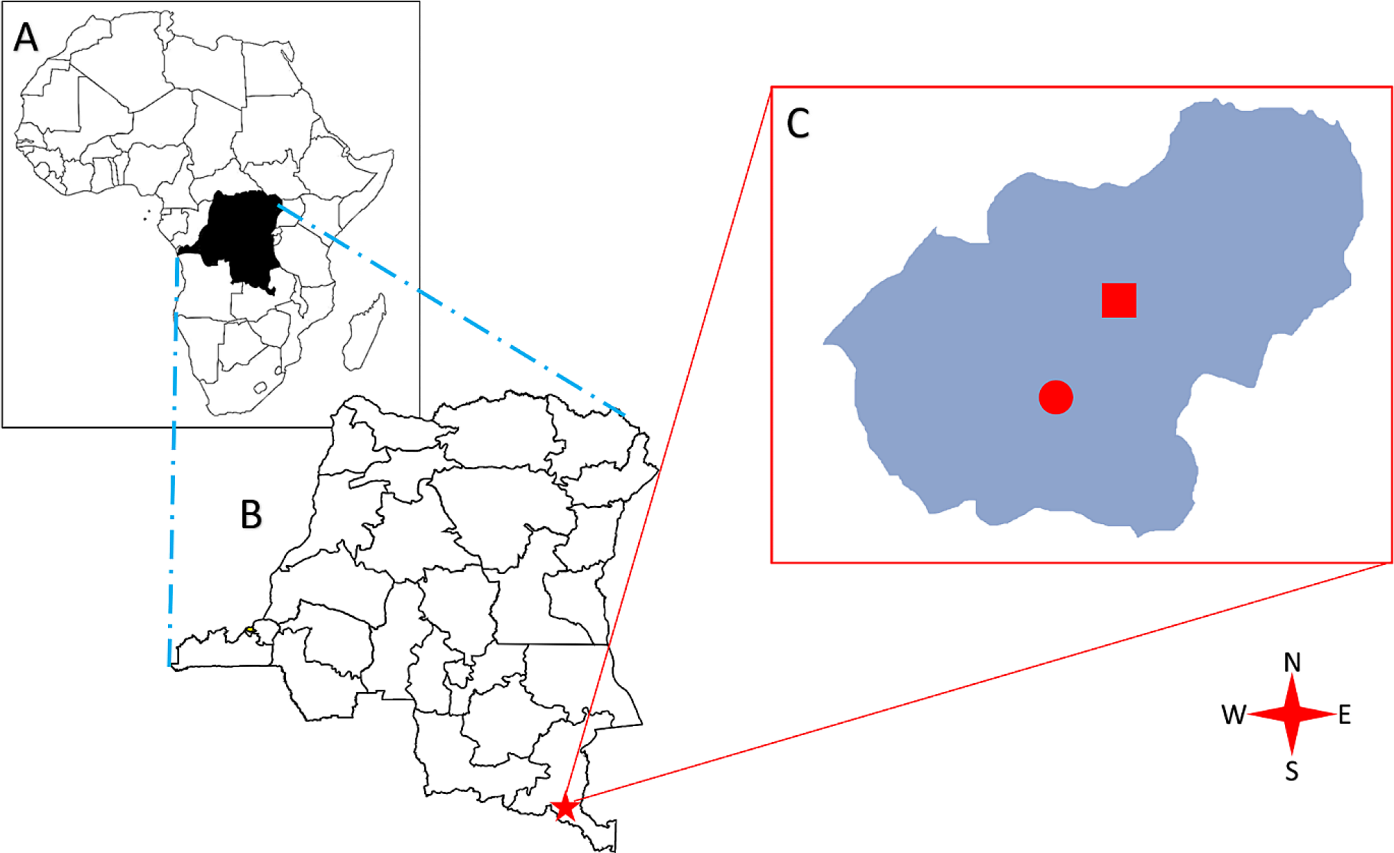
Maps of Africa (**A**), the Republic Democratic of the Congo (**B**), and Lubumbashi city (**C**). The star represents Lubumbashi city, the circle represents the HIV/STI screening and treatment center of the Katuba municipality, and the square represents the HIV/STI screening and treatment center of the Kampemba municipality

Lubumbashi, the capital of Haut-Katanga province, is the second largest city in the DRC and its area is approximately 747 km^2^ (Fig. 1). It is a major economic center due to its role in the mining industry, particularly copper and cobalt. Despite its diverse population, it faces challenges such as urbanization, infrastructure problems, and economic and social disparities. According to estimates for 2024, Lubumbashi’s population will reach 2,933,962 [18]. The town’s centrality in the mining industry attracts a migrant population from other provinces linked to extractive activities, creating a demand for sexual services. Despite the benefits of the mining industry, persistent economic inequality may push some women into sex work as a means of subsistence [18].

**Fig. 2:**
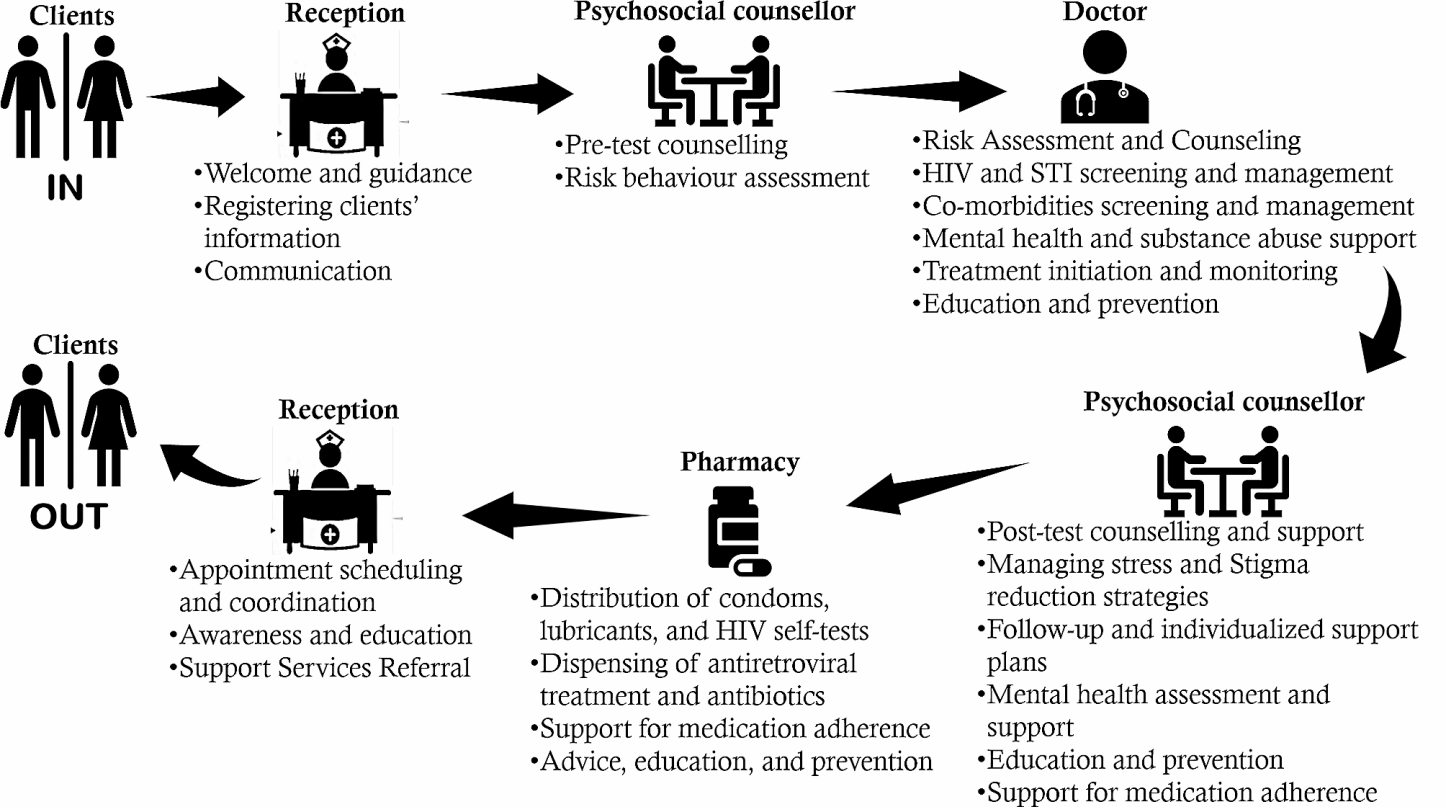
Scheme describing the client circuit in the HIV/STI screening and treatment center for key populations in Lubumbashi, between April 2016 and December 2017

Rapid urbanization and population growth in Lubumbashi have led to increased demand for sexual services, resulting in the rise of sex workers. However, these sex workers often face marginalization, affecting their access to healthcare and safety. In response, two health facilities were established in 2016 in Katuba and Kampemba municipalities to care for HIV and other sexually transmitted infections (STIs) among key populations (sex workers, injecting drug users, men who have sex with men, and transgender people). This study took place in one of these health facilities, which is located in the municipality of Katuba. In this HIV/STI screening and treatment center for key populations, the client circuit is designed to meet the needs of these populations, as illustrated in Fig. 2.

### Study design and population

A cross-sectional study was conducted on 572 FSWs aged 18–24 who visited a HIV/STI screening and treatment center of the Katuba municipality in Lubumbashi, between April 2016 and December 2017. Peer navigators, current or former FSWs, sensitized FSWs from their sex work locations and invited them to visit the center for free healthcare. The study included 572 participants who identified as FSW and met the inclusion criteria of being born biologically female, aged 18–24, and had exchanged regular or occasional sexual acts (such as oral, vaginal, and/or anal intercourse) with anyone other than her established partner in the last six months for something of value (money or/and material goods) that would not have been extended to them by their sexual partners [5, 19]. In the DRC, the legal age for consensual sex is 18 years and over; thus, women under the age of 25 who regard themselves as involved in the sex work are classified as YFSW [4].

Clinical psychologists and doctors conducted face-to-face interviews using structured questionnaires in French or Kiswahili. Interviewees had privacy in the collected rooms. HIV testing was conducted in the doctor’s office after obtaining written informed consent from FSW, with pre- and post-test counseling provided. HIV testing followed the national HIV testing protocol recommending the use of an Alere Determine™ HIV-1/2 Ag/Ab Combo rapid test; if this test is positive, confirmation with a Uni-Gold™ (Trinity Biotech) or VIKIA® HIV1/2 (bioMerieux) rapid test should be done as a second-line test. Individuals reacting to both tests were classified as HIV-positive. For FSWs who tested positive for HIV, antiretroviral treatment was immediately initiated in accordance with the DRC’s national HIV and AIDS management guidelines. All FSWs also received health education and free condoms as shown in Fig. 1.

### Study variables

The dependent variable was HIV status (negative or positive). We used the medical forms and the center’s registers to collect the following independent variables: the participant’s age, the number of years she had been selling sex, the regular consumption of alcohol before sex, the average number of sexual encounters per day, the number of clients per week (paying sexual partners during the previous week), the fact of having had an STI during the previous 12 months (“No” or “Yes”), systematic condom use by the client in the last 12 months (“No” or “Yes”), anal sex in the last 12 months (“No” or “Yes”), forced sex in the last 12 months (“No” or “Yes”). After collecting data from medical forms and the center’s registers, we entered the information into a secure database using Microsoft Excel.

### Statistical analysis

The data collected were recorded in Microsoft Excel and analyzed using STATA version 16. Descriptive analysis was carried out using calculations of proportions for qualitative variables (frequencies and percentages) and medians with interquartile ranges (IQR) for non-normally distributed quantitative variables after verification by Shapiro-Wilk’s test. The Mann-Whitney *U* test was used to compare the medians between HIV positive YFSWs and HIV negative YFSWs. For bivariate analysis, we used the Chi-squared test to check the association between the independent variables and the dependent variable, which was HIV status. To account for possible confounding factors, we entered all independent variables into the final model using multiple logistic regression by the block entry method. We calculated adjusted odds ratios (aOR) with their 95% confidence intervals (95% CI) and considered p-values < 0.05 statistically significant.

### Ethical considerations

The study was approved by the Medical Ethics Committee of the University of Lubumbashi and adhered to ethical guidelines for research in the Democratic Republic of the Congo and the 1964 Declaration of Helsinki. Individual informed consent was not required due to the retrospective secondary analysis of data collected as part of routine care, and patient identifiers were not collected to protect participant anonymity and confidentiality.

## Results

A total of 572 YFSWs were included in the study, of whom 30.8% were aged between 18 and 19, 26.7% between 20 and 21, and 42.5% between 22 and 24. The median age of the YFSWs was 21 years (IQR: 19–24). The median duration of sex work was 1 year (1–3). The majority (76.1%) of YFSWs stated that they regularly consumed alcohol before sex and 78.1% admitted having suffered from an STI in the last 12 months. The median number of sexual encounters per day was 6 (IQR: 3–10). The median number of paying clients per week was 5 (IQR: 3–9). Over the last 12 months, the proportions of YFSWs who reported that they did not systematically use condoms, that they had had anal sex and that they had forced sex were 21.7%, 4.7% and 5.1% respectively (Table 1).

HIV hospital proportion among YFSWs was 3.3% (19/572) with a 95% confidence interval of 2.1–5.1%. The median age of HIV-negative YFSWs was 21 years (IQR: 19–23), while that of HIV-positive YFSWs was 22 years (IQR: 20–23) (*p* = 0.519). HIV infection was not statistically associated with age, duration of sex, work alcohol consumption before sex, number of sexual encounters per day, number of paying clients per week, and having an STI in the last 12 months (*p* > 0.05) (Table 1).

**Fig. 3:**
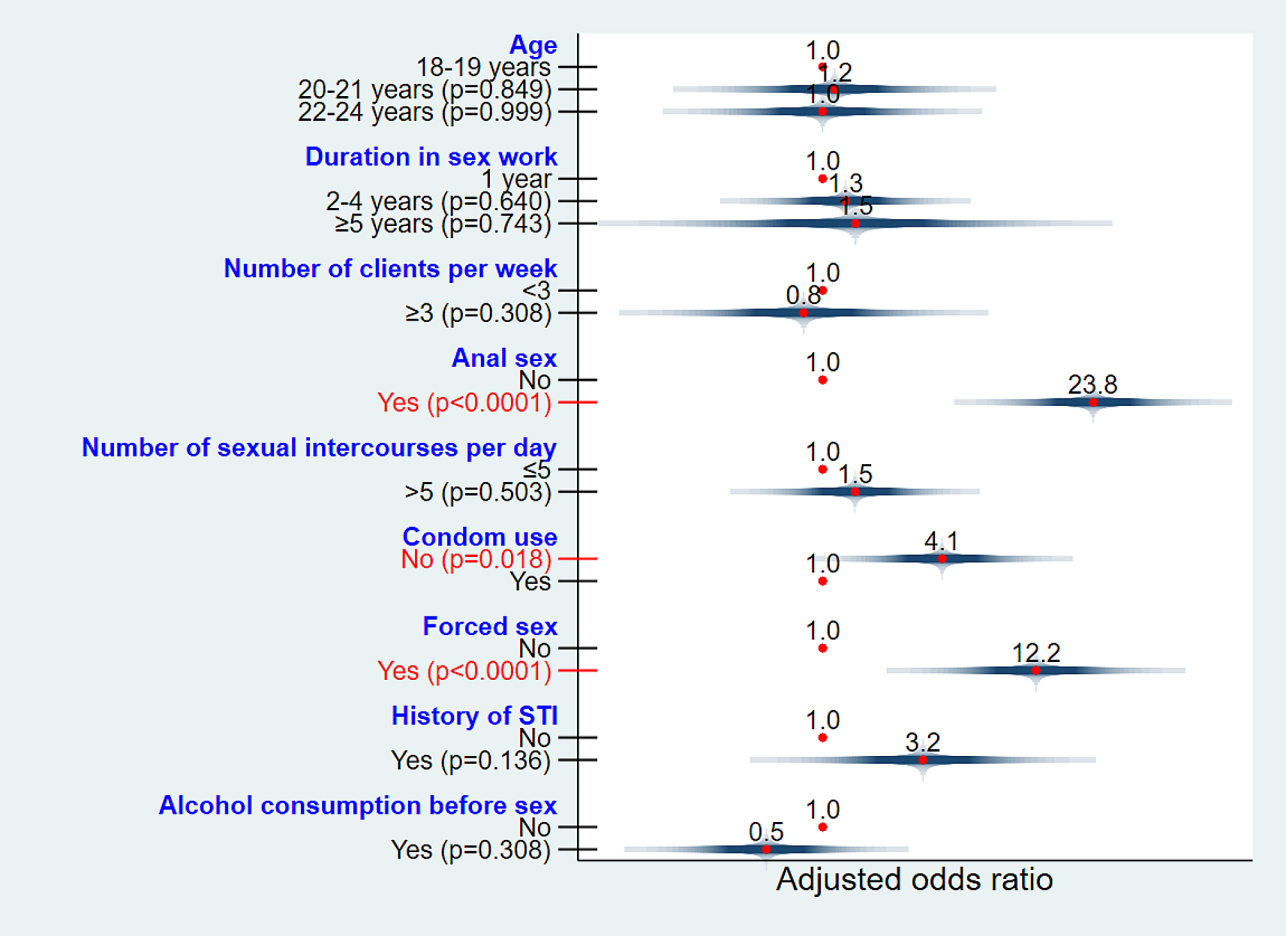
Multiple logistic regression of HIV infection’s determinants among young female sex workers in Lubumbashi, between April 2016 and December 2017

We observed that the odds of being diagnosed with HIV among YFSWs who did not use condoms systematically were significantly higher than among YFSWs who reported using condoms systematically (aOR = 4.1; 95% CI: 1.3–13.0; *p* = 0.018). YFSWs who had forced sex were more likely to be diagnosed with HIV than those who had not (aOR = 12.2; 95% CI: 3.2–46.4; *p* < 0.0001). Anal sex was more susceptible to HIV diagnoses in YFSWs (adjusted OR = 23.8; 95% CI: 6.9–82.4; *p* < 0.0001) (Fig. 3).

**Table 1:** Distribution of age and sexual practices according to HIV status in 572 young female sexual workers in Lubumbashi, between April 2016 and December 2017

Variable	Total (*N* = 572)	HIV status		Unadjusted odds ratio [95% confidence interval]	p-value
		Positive (*n* = 19), n (%)	Negative (*n* = 553), n (%)		
**Age**, *median (interquartile range)*	21 (19–24)	22 (20–23)	21 (19–23)		0.519*
18–19 years	176 (30.8)	4 (2.3)	172 (97.7)	1.0	
20–21 years	153 (26.7)	5 (3.3)	148 (96.7)	1.5 [0.3–7.5]	0.738
22–24 years	243 (42.5)	10 (4.1)	233 (95.9)	1.8 [0.5–8.2]	0.412
**Duration in sex work**, *median (interquartile range)*	1 (1–3)	2 (1–2)	1 (1–2)		0.325*
**Number of sexual intercourses per day**, *median (interquartile range)*	6 (3–10)	6 (3–10)	6 (3–10)		0.951*
**Number of clients per week**, *median (interquartile range)*	5 (3–9)	5 (4–7)	4.5 (3–9)		0.789*
**Alcohol consumption before sex**	435 (76.1)	14 (3.2)	421 (96.8)	0.9 [0.3–2.5]	1.000
**History of sexually transmitted infections**	447 (78.1)	16 (3.6)	431 (96.4)	1.5 [0.4–8.2]	0.777
**No systematic condom use**	124 (21.7)	10 (8.1)	114 (91.9)	4.3 [1.7-10.78]	0.002
**Anal sex**	27 (4.7)	8 (29.6)	19 (70.4)	20.4 [7.4–56.6]	< 0.0001
**Forced sex**	29 (5.1)	6 (20.7)	23 (79.3)	10.6 [3.7–30.5]	< 0.0001

*p-value from the Mann-Whitney U test

## Discussion

This study focuses on understanding the HIV prevalence and its determinants among YFSWs in Lubumbashi. The research provides insight into the complex dynamics surrounding HIV risk factors within the city’s sex work industry, highlighting the nuanced factors that influence HIV transmission. The World Health Organization categorizes the global population’s vulnerability to HIV/AIDS into different groups, with FSWs being a key population susceptible to high HIV infection rates [19]. The study found a 3.3% hospital proportion of HIV infection among YFSWs, higher than the general Congolese population’s 0.7% [13]. However, a Zimbabwean study found a significantly higher HIV prevalence among 2387 YFSWs at 23.6% [4]. These results raise questions about regional determinants and socio-economic factors that could contribute to these variations in HIV prevalences, such as differences in access to health services, sex education, condom availability, and other socio-economic variables.

This study reveals significant variations in HIV hospital proportion rates among YFSWs across different age groups. YFSWs aged 18–19 have a rate of 2.3%, while those aged 20–21 have a rate of 3.3%. The rate increases to 4.1% for those aged 22–24. This indicates a worrying trend of increasing HIV prevalence as YFSWs age, highlighting the need for targeted interventions tailored to each age group. Factors contributing to this trend include prolonged exposure to risky sexual behaviors, unsafe practices, social stigma, and limited access to health services. YFSWs who have been working for several years also face increased challenges due to growing stigmatization, persistent social vulnerability, socio-economic situations, and lack of a social safety net. The Ghanaian study by Guure et al. [5] found variations in HIV prevalence rates between the two age groups studied, with the under-20s having a higher rate of 4.22% and the 20-24s having a slightly lower rate of 2.93%. These differences highlight the need for a comprehensive analysis of socio-cultural contexts, risky sexual practices, and HIV prevention interventions specific to each region.

The study shows that YFSWs engaged in anal sex have a significantly higher risk of being HIV-positive. The risk of HIV transmission through anal sex is 16–18 times greater than penile-vaginal intercourse [20]. Anal intercourse can compromise natural barriers to HIV transmission, causing microtrauma to the rectal mucosa, which is more susceptible to injury than vaginal mucosa [21, 22]. Additionally, the rectum is also rich in immune cells that can be susceptible to HIV infection [22].

Forced sex among YFSWs can lead to increased risk of HIV infection due to barriers such as physical or psychological coercion that sex can compromise the ability to negotiate condom use. Similar to the present study, Sarkar et al. [23] also found that forced sex was significantly associated with HIV infection among FSWs. Forced sex exposes women to high-risk sexual partners, such as those living with HIV who do not know their HIV status. Additionally, AGYW who have been victims of forced sex may develop significant psychological trauma, which may lead them to adopt risky behaviors such as substance use, engagement in unprotected sex, and reduced compliance with HIV prevention measures. A Russian study reported a strong association between drug use, unprotected anal sex and forced sex among FSWs [24].

The isolation and marginalization experienced by FSWs make it difficult for them to negotiate safer sex practices, increasing their risk for HIV infection [25]. Regular condom use significantly reduces the risk of HIV/STIs transmission among FSWs [26, 27]. However, when unprotected sex becomes the norm, the probability of contracting HIV increases considerably. Condom non-use during sex among Ghanaian YFSWs was influenced by higher payments, drug and/or alcohol use, fear of violence sexual, and police harassment [28].

YFSWs’ motivation to engage in risky sexual behavior is influenced by various factors, including economic, social, psychological, and cultural aspects. Economic pressures may lead YFSWs to provide specific sexual services, such as not using condoms and/or anal sex, to receive higher remuneration. Fear of losing clients or rejection may also deter them from refusing specific requests [5]. Lack of adequate sex education and access to accurate information on risky sexual practices can lead to an underestimation of the dangers of sex. Stigmatization and marginalization can also lead YFSWs to adopt risky sexual behaviors to meet clients’ expectations [25]. Psychosocial factors, such as trauma, violence, or poor past treatment, can also influence YFSWs’ sexual choices. The interconnection between unprotected sex, anal sex, and forced sex creates a complex dynamic that amplifies vulnerability and the risk of HIV transmission. This interconnection between these risky sexual behaviors generates a spiral of risk and vulnerability, with each practice reinforcing the others. Anal sex can lead to opportunistic decisions not to use a condom and is often associated with higher financial compensation [29]. Forced sex, by creating situations where control over condom use is limited, increases the risk of HIV transmission and establishes a climate of persistent vulnerability. Targeted interventions such as HIV prevention programs, psychological support services, and awareness-raising campaigns are crucial to break this spiral and improve the overall sexual health of this vulnerable population.

This study investigates risky sexual practices among YFSWs in Lubumbashi. However, it has limitations, including the cross-sectional design that doesn’t establish causal relationships between high-risk sexual practices and HIV infection, and recall bias due to participants’ difficulty remembering past practices. The sample’s representativeness is limited to a single health center, limiting generalizability to all Congolese YFSWs. Socially desirable responses could affect data validity. Despite these limitations, the study has strengths, such as carefully selecting a limited sample, collecting data from participants’ medical records (ensuring clinical accuracy and minimizing recall bias), using robust statistical methods like logistic regression, and highlighting the specific needs of YFSWs, including economic barriers and obstacles to condom use. This study provides a foundation for further research and targeted interventions aimed at reducing risky sexual practices and improving sexual health among YFSWs in Lubumbashi.

## Conclusion

The study reveals a complex link between risky sexual practices, such as unprotected sex, anal sex, and forced sex, and higher HIV prevalence in YFSWs. This is influenced by economic pressure, barriers to condom use, and episodes of forced sex. These factors reinforce vulnerability and increase the risk of HIV transmission. The findings suggest the need for targeted interventions to address vulnerability, such as improving access to condoms, psychological support services, and economic empowerment. These interventions should align with the DRC’s national strategic plan for the HIV response 2023–2027, focusing on prevention, universal access to diagnosis and treatment, strengthening strategic information systems, and creating a supportive environment for HIV prevention and care. Future efforts should focus on increasing engagement with HIV-related services, enhancing accessibility, and eliminating socio-cultural barriers. Further research could explore cultural and social determinants of high-risk sexual practices, longitudinal monitoring, comparative analyses to contextualize findings, intervention studies, collaborative research, and policy analyses to inform improved healthcare access and support.

## Data Availability

The datasets used and/or analysed during the current study are available from the corresponding author on reasonable request.

## Abbreviations

95% CI: 95% confidence interval
AGYW: Adolescent girls and young women
aOR: Adjusted odds ratios
DRC: Democratic Republic of the Congo
FSW: Female sex worker
HIV: Human immunodeficiency virus
IQR: Interquartile range
PEP: Post-exposure prophylaxis
STI: Sexually Transmitted Infection
YFSW: Young female sex worker
